# Construct validity of a continuous metabolic syndrome score in children

**DOI:** 10.1186/1758-5996-2-8

**Published:** 2010-01-28

**Authors:** Joey C Eisenmann, Kelly R Laurson, Katrina D DuBose, Bryan K Smith, Joseph E Donnelly

**Affiliations:** 1 Departments of Kinesiology and Pediatrics & Human Development, Michigan State University, East Lansing, USA; 2 Department of Kinesiology, Illinois State University, Bloomington-Normal, USA; 3 Department of Exercise and Sports Science, East Carolina University, Greenville, USA; 4 Life Span Institute, University of Kansas, Lawrence, USA

## Abstract

**Objective:**

The primary purpose of this study was to examine the construct validity of a continuous metabolic syndrome score (cMetS) in children. The secondary purpose was to identify a cutpoint value(s) for an adverse cMetS based on receiver operating characteristic (ROC) curve analysis.

**Methods:**

378 children aged 7 to 9 years were assessed for the metabolic syndrome which was determined by age-modified cutpoints. High-density-lipoprotein cholesterol, triglycerides, the homeostasis assessment model of insulin resistance, mean arterial pressure, and waist circumference were used to create a cMetS for each subject.

**Results:**

About half of the subjects did not possess any risk factors while about 5% possessed the metabolic syndrome. There was a graded relationship between the cMetS and the number of adverse risk factors. The cMetS was lowest in the group with no adverse risk factors (-1.59 ± 1.76) and highest in those possessing the metabolic syndrome (≥3 risk factors) (7.05 ± 2.73). The cutoff level yielding the maximal sensitivity and specificity for predicting the presence of the metabolic syndrome was a cMetS of 3.72 (sensitivity = 100%, specificity = 93.9%, and the area of the curve = 0.978 (0.957-0.990, 95% confidence intervals).

**Conclusion:**

The results demonstrate the construct validity for the cMetS in children. Since there are several drawbacks to identifying a single cut-point value for the cMetS based on this sample, we urge researchers to use the approach herein to validate and create a cMetS that is specific to their study population.

## Introduction

The constellation of adverse cardiovascular disease (CVD) and metabolic risk factors, which include elevated abdominal obesity, blood pressure, glucose, and triglycerides (TG) and lowered high-density lipoprotein-cholesterol (HDL-C), has been termed the metabolic syndrome [1]. Based on the National Cholesterol Education Program Adult Treatment Program III (ATP II) criteria [1] and the International Diabetes Foundation criteria [2] approximately 35% and 39%, respectively, of U.S. adults possess the metabolic syndrome [3]. In terms of health outcomes, the metabolic syndrome is associated with an increased risk of all-cause mortality, CVD morbidity and mortality, type 2 diabetes, and some cancers [4].

In 1980, Khoury et al. [5] were perhaps the first to show the clustering of CVD risk factors in 6-19 yr old subjects of the Cincinnati Lipid Research Clinic's Princeton Study. In 1987, Smoak et al. [6] also reported the clustering of CVD risk factors in the Bogalusa Heart Study. However, not until the recent medicalization and coining of the term 'metabolic syndrome' had reports indicated the emergence of the metabolic syndrome during childhood and adolescence [7-11]. Data from the United States (U.S.) National Health and Nutrition Examination Survey (NHANES) III (1988-1994) showed that the prevalence rate of the metabolic syndrome was 4% in 12-19 yr old adolescents and 30% in obese adolescents [11]. The prevalence rate increased to 6% (>2 million) in NHANES 1999-2000 [10]. Another paper using the same data (i.e., NHANES III) but different criteria showed that the prevalence rate was nearly 10% [12]. Among 7-to-9 yr old children residing in a rural Midwestern US state, the prevalence rate was 5% [9]. Prevalence rates in children and adolescents have been reported to be 6.5% in northern Mexico [13], 9% in Korea [14], 2% in Turkey [15], and 10% in Quebec, Canada [16].

Since there is no universal definition of the metabolic syndrome in children or adolescence and the prevalence rate is relatively low, several authors [17-27] have derived a continuous score representing a composite CVD/metabolic risk factor profile or index (i.e., the metabolic syndrome score) which has been recommended in a joint statement by the American Diabetes Association and the European Association for the Study of Diabetes [28]. Previous procedures to derive the score include principal component analysis [21,22], Z scores [17,18,21,23-26], and centile rankings [19,20]. However, the validity of these composite scores in children and adolescents has not been examined. A recent paper in adults [29] showed a continuous metabolic syndrome score (cMetS) derived from principal component analysis was higher in adults with the metabolic syndrome and that the score increased progressively with increasing number of adverse risk factors.

Besides validating the cMetS, another related issue is identifying the cMetS that confers increased risk of the metabolic syndrome. In other words, what is the 'optimal' cutoff for interpreting the extent of the cMetS? Recently, receiver operating characteristics (ROC) analysis has been used to determine waist circumference and insulin resistance cutoff values for identifying the metabolic syndrome in youth [30,31]. Based on the increased use and utility of the cMetS in pediatric epidemiological research, the primary purpose of this study was to examine the construct validity of a cMetS in children. The secondary purpose was to identify a cutoff for an adverse cMetS based on ROC analysis.

## Methods

### Subjects

The participants used for this study were part of a physical activity intervention in eastern Kansas called "Physical Activity Across the Curriculum" (PAAC). It should be emphasized that data analysis in this paper is based on baseline measures prior to the intervention. A sub-sample of 2^nd ^and 3^rd ^grade children (ages 7-9 years old) from each school were recruited for additional baseline testing. Inclusion into the sub-sample consisted of the following: 1) both the parent and child gave their written consent and assent, respectively, to participate in baseline testing in accordance with the Human Subjects Committee at the university; 2) the child had to participate in all of the tests (i.e., the child could not choose which tests to complete); and 3) the child did not have insulin dependent diabetes, cardiovascular disease, or any other disease that limited physical activity participation. A total of 852 children volunteered for the additional testing. Due to time constraints all the children could not participate; therefore, a random sample of 495 of these volunteers was selected to participate in the baseline testing. We compared data on the children who signed up to participate but were not randomized to the sub-sample to the 495 selected for subsample data collection. There were no significant differences in weight (p = 0.208) height (p = 0.7523), or BMI (p = 0.1856). There were more males (47% vs.35%), blacks (7 vs. 2%) and Hispanics (10 vs. 4%) in the subsample than those not selected. The differences for gender and race were purposeful since we over-sampled boys and non-whites to ensure a distribution that was similar to the schools.

Of the 495 children that were randomly selected to participate in the baseline testing, 35 did not participate due to absences on the testing day, 9 ate the morning of the blood draw, 8 did not have height and weight data, and 81 had incomplete blood data, leaving 378 children (194 females, 184 males) (272 White, 42 Hispanic, 23 African-American, 9 Asian, 5 Native American, 1 Pacific Islander, 16 more than one race, 10 unknown or not reported) with complete data for statistical analysis. Sample bias was not present, as demographic characteristics were similar between those children who were either included or excluded from the data analyses.

### Measures of the components of the metabolic syndrome

Waist circumference (WC) was measured in duplicate to the nearest 0.1 cm using a Gullick tape measure at the smallest girth around the trunk in the horizontal plane underneath the participant's clothing. There was no difference for inter-tester reliability for waist circumference and the coefficient of variation was 1.65%. Resting blood pressure was measured in duplicate by a trained personnel using a random-zero sphygmomanometer (Hawksley & Sons Ltd., England) according to standard methods [32]. To determine the appropriate cuff size the child's arm circumference was measured. Children rested quietly for 5 minutes prior to measurement. The first and fifth Korotkoff sounds were recorded as systolic and diastolic blood pressure, respectively. Mean arterial pressure (MAP) was calculated using the following formula: MAP = ((systolic blood pressure-diastolic blood pressure)/3)) + diastolic blood pressure). Inter-tester reliability for systolic or diastolic blood pressure was similar and the coefficient of variation for both measures was 5.3%. Blood samples were collected by a trained phlebotomist after an 8-hour fast using standard venipuncture methods. Blood samples were then processed at the study site by centrifuging the samples, placing the serum in pre-labeled vials, storing the samples at -70°C, and shipping the samples to the University of Colorado, Health Sciences Center, Denver, CO for further processing. Blood samples remained stored at -70°C until analyses were conducted. Glucose, total cholesterol, and triglyceride concentrations were measured enzymatically using a Cobas Mira Chemistry System (Roche Diagnostic Systems, Indianapolis, IN). High-density-lipoprotein cholesterol (HDL-C) concentrations were also measured enzymatically using a Cobas Mira Chemistry System (Diagnostic Chemicals Ltd, Oxford, CT). Insulin levels were measured using a radioimmuno assay (Diagnostic Systems Laboratory, Webster, TX). Homeostasis model assessment (HOMA), an indicator of insulin resistance, was calculated as fasting insulin (uU/ml) * fasting glucose (mg/dl)/22.5. The coefficient of variation for all blood measurements was <5% for both inter- and intra- assay quality control.

### Classification of the MetS

Presence of the metabolic syndrome was determined by the age-modified cutpoints of the ATP III metabolic syndrome criteria published previously by Cook et al [11]. Subjects with three or more of the following five risk factors were defined as having the metabolic syndrome: 1) triglycerides ≥110 mg/dl (1.13 mmol/l), 2) HDL cholesterol ≤40 mg/dl (1.04 mmol/L), 3) systolic and/or diastolic blood pressure ≥ 90th percentile for age, sex, and height, based on published reference data [33], 4) fasting plasma glucose ≥110 mg/dl (6.10 mmlo/l), and 5) WC ≥ 90th percentile for age and sex from this sample. The dichotomous metabolic syndrome variable (yes/no) was used in the ROC analysis (see statistical analysis).

### Derivation of the continuous MetS score

The inclusion of the key components (i.e., glucose, lipids, blood pressure, and adiposity) is supported by the results of factor analysis in children and adolescents [16,34-36] which shows the underlying patterns or structure among variables showing high degrees of inter-correlation. Thus, there are common variables that can be used to calculate a cMetS. The cMetS was derived by first standardizing the individual cMetS variables (WC, MAP, HOMA, HDL-C, and TG) by regressing them onto age, sex, and race to account for any age-related differences and sex and race differences. The standardized HDL-C was multiplied by -1 since it is inversely related to metabolic risk. The standardized residuals (Z-scores) for WC, MAP, HDL-C, triglyceride, and HOMA were summed to create the cMetS. These variables were chosen since they represent the same variables (except blood glucose) used in the widely-used National Cholesterol Education Program Adult Treatment Panel III [1] and International Diabetes Federation [2] adult clinical criteria for the metabolic syndrome. HOMA was chosen instead of glucose since most children possess normal fasting glucose and HOMA is related to sophisticated measures of insulin resistance [37]. MAP was used since including both systolic and diastolic would load two blood pressure variables into the calculation, and MAP represents both systolic and diastolic blood pressure. A higher cMetS indicates a less favorable metabolic profile.

### Statistical analysis

The percentage of subjects with varying number of adverse risk factors was calculated by sex and for the total sample. Descriptive statistics for physical characteristics and the individual risk factors were calculated by the number of adverse risk factors (0, 1, 2, 3+) and the total sample. The primary purpose of the study was examined by testing for differences in the cMetS across the number of adverse risk factors (0, 1, 2, 3+) using analysis of variance. For the secondary purpose of the study, ROC analysis was utilized to determine cutoff values that minimize the total number of misclassification errors and to provide an evaluation of the global performance of the cMetS to discriminate between those with or without the MetS. ROC analysis evaluates the performance of any continuous variable to discriminate between two mutually exclusive states of disease [38-40]. By using ROC, information about the agreement of the tests will be provided along with suggested cutpoints for the cMetS. ROC provides measures of sensitivity (Se), specificity (Sp), and the area under the ROC curve (AUC). Se can be defined as the probability of a positive test outcome in an individual who possesses MetS (true-positive) and Sp as the probability of a negative test outcome in an individual without MetS (true-negative). Se and Sp are inversely related depending on the cutpoint and indicate local performance of the recommended cutpoint. The optimal cutoff can be identified as the value where the sum of Se and Sp are maximized. ROC analysis involves the plotting of a curve representing the diagnostic Se (true positive rate) and 1 - Sp (false-positive rate) across a wide range of cutoff values using a diagnostic test (in this case, based on MetS status). The area under this curve can be utilized as a measure of the global accuracy of a diagnostic test [40]. More specifically, AUC relates to the overall ability of using the cMetS to discriminate between those with and without MetS. In this analysis, AUC can be considered equivalent to the probability that a randomly drawn individual from the MetS reference has a higher cMetS than a child randomly drawn from the non-MetS sample. It has been suggested that the AUC be interpreted according to the following guidelines: non-informative/test equal to chance (AUC = 0.5), less accurate (0.5 < AUC ≤ 0.7), moderately accurate (0.7 < AUC ≤ 0.9), highly accurate (0.9 < AUC ≤ 1.0), and perfect discriminatory tests (AUC = 1.0) [41].

## Results

Table 1 shows the number and percentage of subjects by the number of adverse risk factors. Overall, about half of the subjects did not have any adverse risk factors and in turn, the other half possessed at least 1 adverse risk factor. About 5% possessed the metabolic syndrome (≥3 risk factors). No children possessed all five adverse risk factors. There were no significant sex differences in the % of subjects possessing a given number of adverse risk factors or in the occurrence of any given risk factor. Elevated BP was the most common risk factor (37%) and abnormal fasting glucose was the least common (1%)(Table 2). Although not included as a component of the MetS, it should be noted that 44% of the sample was either overweight or obese.

**Table 1:** Percentage of adverse components of the metabolic syndrome in boys and girls.

# of risk factors	Boys (n/%) N = 184	Girls (n/%) N = 194	Total sample (n/%) N = 378
0	101 (54.5%)	91 (46.9%)	192 (50.7%)
1	62 (33.7%)	68 (35.0%)	130 (34.4%)
2	12 (6.5%)	26 (13.4%)	38 (10.0%)
3	8 (4.3%)	7 (3.6%)	15 (4.0%)
4	1 (0.5%)	2 (1.0%)	3 (0.8%)
5	0 (0%)	0 (0%)	0 (0.0%)

**Table 2:** Physical characteristics of the total sample and by the number of adverse risk factors.

Number of risk factors					
	0	1	2	3+	Total sample
N	192	130	38	18	378
Age (yrs)	7.7 (0.7)	7.7 (0.7)	7.9 (0.6)	7.7 (0.5)	7.7 (0.6)
Height (cm)	129.7 (6.5)	129.6 (6.4)	133.2 (6.0)	135.0 (7.7)	130.3 (6.6)
Mass (kg)	28.2 (4.6)	29.7 (6.3)	38.4 (10.0)	46.4 (14.6)	30.6 (8.1)
BMI (kg/m^2^)	16.7 (1.7)	17.6 (2.8)	21.4 (4.3)	25.0 (4.7)	17.9 (3.4)
% overweight	-	-	-	-	21%
% obese	-	-	-	-	23%
Body fat (%)	17.5 (5.5)	19.4 (6.8)	27.3 (8.4)	35.6 (9.8)	20.0 (7.9)
WC (cm)	56.9 (4.3)	58.2 (6.6)	66.8 (10.5)	78.6 (13.4)	59.4 (8.4)
% elevated WC	-	-	-	-	10%
SBP (mm Hg)	101.1 (7.2)	109.1 (10.0)	111.8 (9.0)	116.0 (7.3)	105.6 (9.7)
DBP (mm Hg)	63.2 (5.4)	70.7 (8.0)	73.6 (8.8)	73.7 (9.6)	67.4 (8.2)
% elevated BP	-	-	-	-	37%
MAP (mm Hg)	75.8 (4.9)	83.5 (7.0)	86.4 (7.2)	87.8 (7.4)	80.1 (7.5)
Glucose (mg/dl)	78.3 (7.0)	80.8 (6.3)	80.4 (12.0)	81.1 (8.4)	79.5 (7.6)
% elevated glucose	-	-	-	-	1%
Insulin (uU/ml)	5.5 (5.0)	7.6 (8.2)	11.0 (12.2)	12.3 (11.0)	7.1 (7.7)
HOMA	1.08 (1.07)	1.55 (1.77)	2.44 (3.65)	2.52 (2.55)	1.44 (1.86)
TC (mg/dl)	183.4 (32.1)	178.9 (28.3)	179.7 (33.4)	187.7 (25.2)	181.7 (30.7)
LDL-C (mg/dl)	113.1 (29.0)	108.6 (22.3)	110.2 (27.5)	118.3 (22.3)	111.5 (26.4)
HDL-C (mg/dl)	57.9 (10.6)	55.9 (11.6)	49.2 (9.4)	43.1 (8.1)	55.6 (11.4)
Low HDL	-	-	-	-	5%
TG (mg/dl)	62.4 (16.8)	72.0 (25.0)	101.5 (28.4)	131.3 (34.4)	72.9 (28.1)
Elevated TG	-	-	-	-	18%

Values are mean (SD) and % where indicated.

Descriptive statistics for the physical characteristics and individual risk factors by the number of adverse risk factors are presented in Table 2. As would be expected based on the definition of the MetS, there was a general trend for those with more adverse risk factors to be bigger and possess more adverse levels of the risk factors.

**Figure 1:**
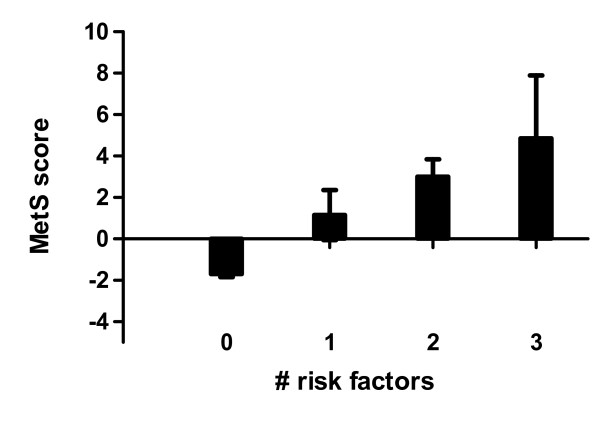
**Metabolic syndrome score by the number of adverse risk factors**.

Figure 1 shows the graded relationship between the cMetS and the number of adverse risk factors. The cMetS is lowest in the group with no adverse risk factors (-1.59 ± 1.76) and highest in those possessing the metabolic syndrome (≥3 risk factors) (7.05 ± 2.73). The mean cMetS for the total sample is 0.00 ± 3.0.

**Figure 2:**
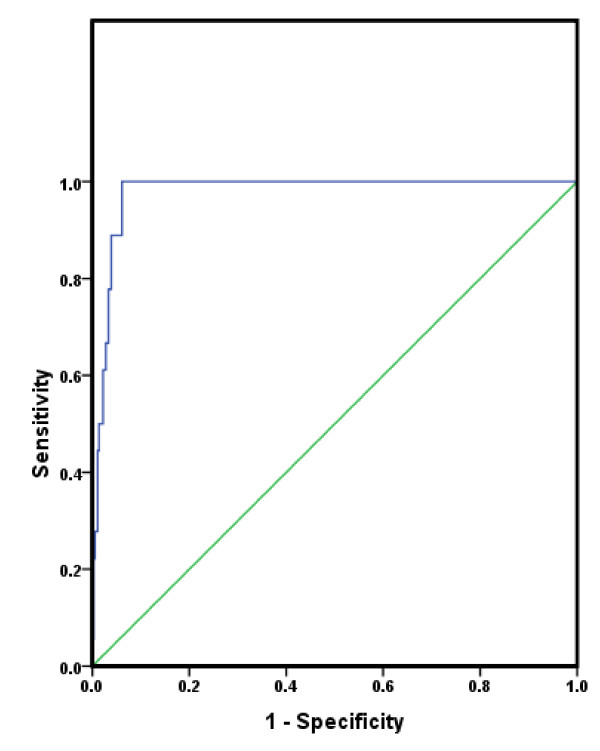
**Receiver operating characteristic curve for the continuous metabolic syndrome score as a predictor of metabolic syndrome among 7-9 year old boys and girls**. AUC = 0.978 (0.957-0.990, 95% confidence intervals).

According to the ROC curve analysis (Figure 2), the cutoff level yielding the maximal sensitivity and specificity for predicting the presence of the metabolic syndrome was a cMetS of 3.72. The sensitivity and specificity using this cutoff value were 100% and 93.9%, respectively. The AUC was 0.978 (0.957-0.990, 95% confidence intervals) (*p *< 0.001). Other cutpoint values and their corresponding sensitivity and specificity are provided in Table 3 for interpretation by the reader.

**Table 3:** Sensitivity and specificity of the continuous metabolic syndrome score to identify children with the metabolic syndrome.

Criterion	Sensitivity (95% CI)	Specificity (95% CI)
1.00	100 (81.3-100.0)	75.14 (70.3-79.5)
1.50	100 (81.3-100.0)	80.83 (76.4-84.8)
2.00	100.0 (81.3-100.0)	84.44 (80.3-88.0)
2.50	100.0 (81.3-100.0)	87.2 (83.3-90.5)
3.00	100.0 (81.3-100.0)	90.28 (86.7-93.1)
3.50	100.0 (81.3-100.0)	92.50 (89.3-95.0)
4.00	88.89 (65.2-98.3)	93.89 (90.9-96.1)
4.50	88.89 (65.2-98.3)	95.56 (92.9-97.4)
5.00	88.89 (65.2-98.3)	95.83 (93.2-97.6)

## Discussion

As previously shown in this sample [9], approximately 5% of 7-9 yr olds possess the metabolic syndrome based on the criteria of Cook et al. [11]. Again, it should be emphasized that there is no universal definition of the metabolic syndrome in children and adolescents and that the prevalence is relatively low. Thus, both of these factors limit the ability to conduct epidemiological studies examining various genetic and environmental risk factors for the metabolic syndrome or the clustering of risk factors in youth. Therefore, we [23-26,42] and others [17,18] have constructed a cMetS for investigations in pediatric epidemiology and medicine. An advantage to a cMetS is that it is statistically more sensitive and less error prone by comparison to the dichotomous approach [18,43]. Several researchers have utilized the cMetS in pediatric epidemiological research. For example, results from the Corpus Christi Child Heart Study indicated higher cMetS in Mexican-American boys and girls compared to White boys and girls [21]. Recent studies also show that habitual physical activity and aerobic fitness are inversely associated with the cMetS [17,18,23,24,26,42], and that aerobic fitness attenuates the cMetS among high fat children and adolescents [24,26,42]. The cMetS has also been shown to track from childhood/adolescence into young adulthood [19,20,22,25]. This latter finding holds importance to the predictive utility of the cMetS on adult disease outcomes.

To date, this is the first study to validate the cMetS score in children. The results indicate a clear graded relationship between the cMetS and the number of adverse risk factors with those possessing ≥3 risk factors (i.e., metabolic syndrome) having the highest cMetS. Furthermore, results of the ROC analysis identified a cMetS of 3.72 as the optimal cutpoint which confers an increased risk of MetS in this sample of 7-9 year old children. The AUC for this index was 98%, which indicates a cMetS of 3.72 is highly accurate at detecting the presence of MetS in this sample. The sensitivity and specificity using this cutoff value were 100% and 93.9%, respectively. In other words, none of the children with metabolic syndrome had a cMetS score lower than 3.72 and only 6% of the children without metabolic syndrome had a score higher than 3.72. This cutoff value also approximates the mean value for subjects possessing 2 adverse risk factors (Table 2). However, other cMetS indicated in Table 3 should also be considered since they also possess high values for sensitivity and specificity. It should also be considered that there may be varying levels of increased risk for MetS (e.g., low, moderate, high) based on the cMetS, particularly since other scores showed good sensitivity and specificity. Hence, cMetS can be used as a preventative measure rather than a diagnostic tool. However, it is important to note that there are several drawbacks to identifying cut-point value(s) for the cMetS based on this sample, and thus we urge researchers to use the approach herein to validate and create a cMetS that is specific to their study population. Some of these limitations are discussed below. First, the cMetS is sample-specific. Second, the cut-off points used herein, e.g., the Cook criteria does not have a solid clinical or health-related outcome basis. Third, the cMetS is based upon the assumption that each selected variable is equally important in defining CVD risk, although a child's being defined as at risk does not necessarily mean that the child has high levels of CVD risk factors per se.

A similar study in adults has also validated the cMetS. In a randomly selected sample of 18- to 75-year-old (mean age = 46 yrs) Flemish adults (571 men and 449 women), the cMetS, determined by principal component analysis, was significantly higher in subjects with cMetS as defined by the International Diabetes Federation (men [12.8%]: 2.03 ± 1.00, women [>8.5%]: 2.63 ± 1.28) versus subjects without (men: -0.30 ± 1.21, women: -0.24 ± 1.16). Moreover, cMetS increased progressively with increasing numbers of risk factors in men (0 risk factors: -1.21 ± 0.96; 1 risk factor: -0.26 ± 0.87; 2 risk factors: 0.67 ± 0.84; 3 risk factors: 1.76 ± 0.73; and 4 risk factors: 3.04 ± 0.94) and women (0 risk factors: -0.96 ± 0.79; 1 risk factors: 0.16 ± 0.82; 2 risk factors: 1.21 ± 0.82; 3 risk factors: 2.17 ± 0.81; and 4 risk factors: 4.09 ± 0.99) [29]. These findings are similar to those reported here and support the use of the cMetS in epidemiological analyses.

As previously mentioned, one limitation to this study is that the MetS criteria - e.g., the Cook criteria - have no clinical or health-related outcome basis. Recently, age-specific criteria for the metabolic syndrome were developed using the LMS statistical technique in 12-19 yr old subjects participating in the NHANES [44]. Unfortunately, we could not use these values since our subjects were 7-9 yr old. The age of the subjects is both a limitation and strength of the study. In terms of limitations, the cMetS needs to be validated in older children and adolescents. Another limitation is that the cMetS is sample specific. If epidemiological studies are to use the cMetS, a standardized method of calculating the score may prove beneficial for comparing studies. An alternative approach to the sample-specific Z score approach highlighted here may be to compare individual risk factor value to the population median or clinical cut-points such as those recently published [44].

In conclusion, this paper shows the convergent validity for the cMetS that is becoming widely used in pediatric epidemiological research. Furthermore, researchers can use the approach herein to validate and create cMetS that are specific to their study populations. It is recommended that age-specific criteria for the metabolic syndrome such as those recently developed for 12-19 yr olds be used as a criterion in future studies and that such criteria be developed in younger children as well.

## Competing interests

The authors declare that they have no competing interests.

## Authors' contributions

JCE was responsible for the conception of the research question, analysis and interpretation of data; and writing the manuscript. KRL has made contributions to the analysis and interpretation of data, and assisted in drafting the manuscript and revising it critically for important intellectual content. KDD was responsible for conception of the research question, interpretation of data, and assisted in drafting the manuscript and revising it critically for important intellectual content. BKS was responsible for data collection and provided critical feedback to the manuscript for important intellectual content. JED was responsible for acquisition of funding, coordinating data collection, and provided critical feedback to the manuscript for important intellectual content. All authors read and approved the final manuscript.
